# The effects of intranasal oxytocin on black participants’ responses to outgroup acceptance and rejection

**DOI:** 10.3389/fpsyg.2022.916305

**Published:** 2022-08-18

**Authors:** Jiyoung Park, Joshua Woolley, Wendy Berry Mendes

**Affiliations:** ^1^Department of Psychology, University of Texas at Dallas, Richardson, TX, United States; ^2^Department of Psychiatry, University of California, San Francisco, San Francisco, CA, United States

**Keywords:** attributional ambiguity, social acceptance, social rejection, intergroup trust, oxytocin, social salience, interracial

## Abstract

Social acceptance (vs. rejection) is assumed to have widespread positive effects on the recipient; however, ethnic/racial minorities often react *negatively* to social acceptance by White individuals. One possibility for such reactions might be their lack of trust in the genuineness of White individuals’ positive evaluations. Here, we examined the role that oxytocin—a neuropeptide putatively linked to social processes—plays in modulating reactions to acceptance or rejection during interracial interactions. Black participants (*N* = 103) received intranasal oxytocin or placebo and interacted with a White, same-sex stranger who provided positive or negative social feedback. After positive feedback, participants given oxytocin (vs. placebo) tended to display approach-oriented cardiovascular responses of challenge (vs. threat), exhibited more cooperative behavior, and perceived the partner to have more favorable attitudes toward them after the interaction. Following negative feedback, oxytocin reduced anger suppression. Oxytocin did not modulate testosterone reactivity directly, but our exploratory analysis showed that the less participants suppressed anger during the interaction with their partner, the greater testosterone reactivity they displayed after the interaction. These results survived the correction for multiple testing with a false discovery rate (FDR) of 20%, but not with a rate of 10 or 5%. Discussion centers on the interplay between oxytocin and social context in shaping interracial interactions.

## Introduction

Social belongingness is a fundamental human need (Baumeister and Leary, 1995); individuals strive to connect with others and gain social approval, and when such a need is met, the resulting sense of acceptance can lead to a variety of positive psychological and biological outcomes (Crocker et al., 1993; Dickerson et al., 2004). Given the significance of social acceptance, however, it would seem puzzling that research finds ethnic/racial minoritized individuals sometimes react *negatively* to social acceptance by White individuals, resulting in lowered self-esteem, feelings of depression, and threat (Crocker et al., 1991; Hoyt et al., 2007; Mendes et al., 2008).

Why might minoritized individuals show these paradoxical responses? One possibility for such reactions might be their lack of trust in the genuineness of White partners’ positive evaluations. Minorities may perceive the positive feedback to be motivated by White partners’ external concern to avoid appearing prejudiced to others, and thus, disingenuous (Crocker and Major, 1989; Major and O’Brien, 2005). The suspicion about the motives underlying positive responses may, in turn, undermine benefits typically associated with social acceptance. Here, we attempt to examine if intranasal oxytocin would modulate affective and social processes stemming from intergroup acceptance (vs. rejection), potentially *via* promoting prosocial outcomes, such as intergroup trust.

Oxytocin is a neuropeptide that has been implicated in the regulation of a wide range of social behavior—both prosocial and antisocial—depending on social contexts (Bartz et al., 2011; Olff et al., 2013; Shamay-Tsoory and Abu-Akel, 2016). In particular, when administered in situations that involve *positive* social interactions, oxytocin has been shown to increase affiliative motive and prosocial behaviors (see Macdonald and Macdonald, 2010; Striepens et al., 2011 for reviews). Thus, the goal of the present research was to examine whether and how intranasal oxytocin (vs. placebo) influences Black participants’ physiological, affective, and behavioral responses to receiving positive (or negative) social feedback from a White interaction partner.

### Paradoxical responses to social acceptance

Substantial evidence has accumulated suggesting that social acceptance from majority group members directed at minorities can engender negative consequences. For example, Crocker et al. (1991) found that after receiving positive interpersonal feedback from a White partner, Black participants showed reductions in their self-esteem, particularly when they had reason to attribute the feedback as stemming from their race—that is, when they believed their partner knew their race. Receiving positive feedback did not reduce self-esteem when participants thought their partner was unaware of their race. Similarly, Hoyt et al. (2007) found that Latin Americans who attributed White partners’ positive behaviors to their race experienced lower self-esteem compared to those who did not make such attributions. Mendes et al. (2008) extended these findings by examining physiological mechanisms underlying minorities’ reactions to outgroup acceptance. Following positive feedback from White partners, Black participants exhibited cardiovascular responses characteristic of threat (less cardiac efficiency and vasoconstriction), whereas those receiving positive feedback from same-race partners showed challenge reactivity (increased cardiac efficiency and vasodilation). Importantly, the deleterious effects of positive feedback were only evident among Black participants; White participants responded positively to positive feedback, regardless of whether their partner was the same- or different-race.

What accounts for these paradoxical responses? Attributional ambiguity theory suggests that minorities might doubt the motives underlying positive feedback from White partners and distrust the authenticity of the feedback (Crocker and Major, 1989; Major and O’Brien, 2005; Major et al., 2016). Because of cultural and legal prohibition against expression of prejudice in current U.S. society, many White individuals are concerned about appearing racist (Plant and Devine, 2003). They might be strongly motivated to regulate their actions not to display any signs of racial bias, in some cases, by over-correcting—that is, acting overly friendly toward minorities (Harber et al., 2010; Mendes and Koslov, 2013). As a result, minorities are likely to experience considerable attributional ambiguity about the true intentions behind White individuals’ positive treatment directed toward them. Initially, minorities may be motivated to believe that the positive behaviors were driven by genuine liking or respect (e.g., Sinclair and Kunda, 2000). However, they may subsequently engage in additional attributional processing and adjust the initial judgment by considering the possibility that the positive behaviors were driven by European Americans’ external concerns over appearing prejudiced. The uncertainty arising from the conflict between these two cognitions might in turn create deleterious reactions (van den Bos, 2009). For example, when minorities’ uncertainty about the motives underlying White people’s positive behaviors were assessed with the Suspicion of Motives Index (SOMI; Major et al., 2013), those who were more suspicious about White people’s motives were more accurate at detecting their external motivation to appear non-prejudiced (LaCosse et al., 2015). Moreover, highly suspicious individuals react more negatively to White people’s positive behaviors, for example, with heightened threat vigilance, elevated stress responses, and decreased self-esteem (see Kunstman and Fitzpatrick, 2018 for review).

If the lack of trust is the underlying mechanism of minorities’ negative reactions, it may then be anticipated that in conditions where suspicion is eliminated, and thus, trust can be enhanced, minorities should react more favorably to the positive feedback because the feedback would be attributionally less ambiguous under such conditions. As an initial attempt to test this idea, we used a pharmacological intervention with intranasal oxytocin to examine whether this hormone would promote positive outcomes in the context of positive (vs. negative) interactions, such as intergroup trust.

### Oxytocin and social processes: The social salience hypothesis

Earlier work in this area focused on the prosocial effects of oxytocin. Several studies reported that oxytocin facilitates affiliative prosocial behaviors, such as trust, cooperation, empathy, and generosity (e.g., Kosfeld et al., 2005; Hurlemann et al., 2010; Arueti et al., 2013). An initial study showed that participants given intranasal oxytocin, relative to placebo, gave more money to others in a trust game (Kosfeld et al., 2005; but see also Nave et al., 2015; Declerck et al., 2020 for recent failed replications). This finding was conceptually replicated by Baumgartner et al. (2008), who further showed that the prosocial effects of oxytocin were explained by reductions in activity in the amygdala, thereby suggesting that oxytocin may promote trust by reducing fear and anxiety about potential negative consequences of social interaction, such as betrayals (see Churchland and Winkielman, 2012 for similar argument).

More recent work, however, suggests that the effects of oxytocin are more nuanced than are often claimed by showing that many of the previously reported prosocial effects of oxytocin are context-dependent (Shamay-Tsoory et al., 2009; Mikolajczak et al., 2010; DeWall et al., 2014). For example, increasing evidence suggests that oxytocin facilitates prosociality only in contexts relatively free of negative interpersonal cues. Oxytocin promotes trust toward a partner who is perceived as trustworthy (vs. untrustworthy; Mikolajczak et al., 2010), and only toward ingroup members, but not toward outgroup members, when the interaction involves intergroup competition where negative aspects of outgroup members are likely made salient (De Dreu et al., 2010; De Dreu, 2012). Moreover, in the presence of negative interpersonal cues, oxytocin even facilitates antisocial reactions, such as experiences of envy and schadenfreude in response to monetary loss in a competitive game (Shamay-Tsoory et al., 2009) and aggressive behaviors following provocation (Ne’eman et al., 2016).

To reconcile these disparate findings, it has been proposed that oxytocin modulates attention-orienting responses to contextual social cues, thereby enhancing perceptual salience and processing of these cues (i.e., the social salience hypothesis; Bartz et al., 2011; Olff et al., 2013; Shamay-Tsoory and Abu-Akel, 2016). According to this view, oxytocin can produce a wide variety of responses—both positive and negative—depending on the available social stimuli in a given context. Oxytocin may promote prosociality when the context involves positive interpersonal cues (Mikolajczak et al., 2010), whereas it is likely to facilitate competitive or aggressive behaviors when the context involves negative interpersonal cues (De Dreu et al., 2010; DeWall et al., 2014; Ne’eman et al., 2016). The enhanced salience of social cues, enabled by oxytocin, may in turn, motivate individuals to make an immediate reaction based on intuitive processing in response to imminent situational contingencies. In support of this formulation, recent evidence suggests that oxytocin facilitates intuitive and spontaneous actions than deliberate and controlled responses (Ma et al., 2015; Ten Velden et al., 2016).

Taken together, this body of work suggests that oxytocin may play a different role in interracial interactions depending on available social cues, such as the type of feedback people receive. We predicted that oxytocin would enhance affiliative motive and prosociality when positive social cues are salient—that is, when Black participants receive positive feedback from the White partner. The initial attention to the positive feedback, if enhanced under the condition of oxytocin, can bolster and validate the feedback while inhibiting biased reactions based on additional attributional information. As a consequence, Black participants in this condition would react more favorably to the positive feedback, with increased liking, approach tendencies, and cooperation. In contrast, social rejection from an outgroup member typically engenders antagonistic reactions, such as aggression and anger (Major et al., 2002; Mendes et al., 2008). We predicted that oxytocin might amplify these negative emotional reactions, due to the enhanced perceptual sensitivity to the negative social cue (i.e., the outgroup member as a source of rejection).

It is important to note, though, that while there has been a surge of research on the role that oxytocin plays in human behaviors over the past two decades, there has also been a fair amount of research questioning the role and reliability of oxytocin effects on social behavior. Critical reviews have questioned the affective specificity of oxytocin, the extent to which intranasal oxytocin has direct effects on the central nervous system, and whether there is a strong foundation of data supporting the conclusions (e.g., Nave et al., 2015; Declerck et al., 2020; Mierop et al., 2020). To address some of these criticisms, we took seriously the role of social context to examine the effects of intranasal oxytocin on social behavior using face-to-face interactions by following best practices in oxytocin research available at the time.

### Research overview

Our goal was to examine the role that intranasal oxytocin plays in modulating minorities’ responses to outgroup acceptance or rejection by adopting a paradigm by Mendes et al. (2008). This allowed us to conceptually replicate some of their main findings and extend them by including intranasal administration of oxytocin vs. placebo as an additional factor during interracial interactions in a placebo controlled, double-blind experiment.

We hypothesized that oxytocin would lead Black participants to react more favorably to the positive feedback by the White partner, with increased approach motivation, cooperation, and liking. These outcomes were assessed based on participants’ physiological, behavioral, and affective responses. First, participants’ motivational states of approach (vs. avoidance) were captured based on challenge vs. threat patterns of cardiovascular responses (Blascovich and Mendes, 2000). Second, we included a public goods provision task to measure participants’ cooperative behavior. Third, participants’ partner perceptions and affective responses were assessed with self-report measures.

In contrast, we hypothesized that the prosocial effects of oxytocin would be diminished in the negative feedback condition. Instead, we predicted that oxytocin might amplify antagonistic reactions typically following outgroup rejection, such as anger responses. We tested this hypothesis in two ways. First, we administered the Anger Expression Scale (AX; Spielberger et al., 1986) to assess the extent to which participants expressed, suppressed, or controlled their angry feelings during the interaction with their partner. Second, to alleviate concerns regarding self-presentational issues, we also measured testosterone responses that are often associated with experiences of anger and dominance (e.g., Mehta et al., 2008).

## Materials and methods

### Participants

One hundred and six Black Americans between the ages of 20 and 35 (61 women; 45 men, *M*_age_ = 25.31, *SD*_age_ = 4.83) were recruited from the community. The study took place in San Francisco, which has less than 6% Black/African American residents, underscoring the social context of individuals as numerical minorities. We planned to recruit a minimum of 100 participants, with 25 participants per condition. This sample size was determined *a priori* based on previous studies that involved a similar pharmacological intervention and physiological assessments (e.g., Kubzansky et al., 2012; Human et al., 2018). A discussion of the sample size and associated power is included later in the paper (see Robustness Checks section). Prior to the lab session, participants were screened for exclusion criteria, including (a) current or past psychiatric disorder (e.g., clinical depression or clinical anxiety), (b) significant medical illnesses (e.g., heart arrhythmia or hypertension), (c) pregnancy, and (d) obesity (body mass index > 35). Before coming into the lab, participants were asked to abstain from caffeine, alcohol, and exercise for at least 2 h. They were compensated $50 and received additional $17 bonus (see below).

### Procedure

**FIGURE 1:**
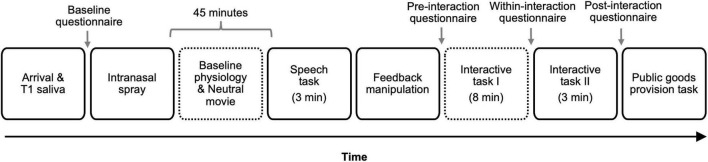
Study timeline. T1 indicates Time 1. The number in parenthesis indicates the duration of the task for tasks with fixed duration. Time 2 [T2] and Time 3 [T3] saliva samples were obtained 18 and 33 min following the onset of the first interactive task (i.e., taboo game), respectively. Dotted outlines indicate the times when cardiovascular responses were assessed. Reactivity indices were computed on three physiological parameters (heart rate, pre-ejection period, and cardiac output) by subtracting participants’ baseline responses obtained during the last minute of the initial resting period from the physiological responses obtained during the first segment of the interactive task.

The study involved a 2 Intranasal spray (oxytocin vs. placebo) × 2 Feedback (positive vs. negative) between-participants, double-blind, placebo-controlled design. The placebo conditions offered an opportunity to conceptually replicate Mendes et al. (2008). All procedure and materials were approved by the Institutional Review Board at the study site. The study consisted of six phases and took approximately 2 h. See Figure 1 for the timeline of the study.

#### Phase 1: Arrival and baseline saliva assessment

To minimize the effects of circadian fluctuations in testosterone levels (Touitou and Haus, 2000), participants were scheduled to come to the lab between 12:00 pm and 5:30 pm. After providing informed consent, female participants were asked to provide a urine sample for a pregnancy test and were excused from participation if the tests were positive. Participants then provided a 1.5 mL saliva sample that served as baseline testosterone assessment (Time 1 [T1]). Participants were instructed to expectorate into a sterile polypropylene microtubule (IBL tubes). Right afterward, participants completed the *baseline questionnaire* to assess their baseline affective states.

#### Phase 2: Intranasal spray and baseline physiological recording

Next, participants self-administered a nasal spray containing 40 international units (IU) of oxytocin (syntocinon spray, Novartis) or placebo (containing all inactive ingredients except the neuropeptide) in the presence of the study MD (second author) or a trained project director (see Woolley et al., 2016; Petereit et al., 2019; Thorson et al., 2021; for a similar procedure). After the administration of the intranasal spray, we attached sensors for physiological measurement and participants’ physiological responses were recorded for 5 min while they sat quietly (see Cardiovascular Responses section for more details on physiological assessment). Prior work suggests that intranasal oxytocin begins to exert an influence on behavioral and physiological responses at least 30 min after the administration and last for a minimum of 90 min (Norman et al., 2011). Thus, after the baseline recording, participants were asked to complete a 30-min relaxation period that included watching an emotionally neutral film (a documentary about hiking the Appalachian Trail). Approximately 45 min after the intranasal spray, the first task of the study (i.e., speech task, see below) was introduced. From the first active task to the last task occurred within a 90-min time frame.

#### Phase 3: Speech task

At least 45 min following the intranasal spray, participants were given instructions for the speech task. From this point forward, we adopted the protocol used in Mendes et al. (2008).^1^ Participants were told that they would interact with another participant (i.e., a confederate), who was in a different lab room. All participants verbally consented to continue with this part of the experiment and were then introduced to a gender-matched, White confederate. We made an audiovisual connection between the two experiment rooms so that the participant and the confederate could see and hear each other over large television monitors (42”).

After the brief introduction, the participant and the confederate were informed that they would be randomly assigned to one of two roles—a performer or an evaluator—for the upcoming speech task. The participant was asked to select one of two cards (A or B) from a random assignment box and was told that the person who chose card A (or B) would be assigned to the performer condition while the person who chose card B (or A) would be assigned to the evaluator condition. Regardless of the card choice, the participant was always the performer who was told to deliver a speech on the topic of “Why I make a good friend” for 3 min while their partner listened to the speech.

After providing speech instructions, we disconnected the audiovisual connection between the two rooms so that the confederate could not see or hear the participant’s speech; however, the participant was told that the connection was still on and their partner could see and hear their speech. After a 1-min preparation period, the participant delivered the speech for 3 min.

#### Phase 4: Feedback manipulation

After the speech, the experimenter returned to the room and asked participants to answer several questions on the computer about their experience during the speech and explained to them that some of this information would be electronically exchanged with their partner. After completing the questionnaire, participants were asked to click “SEND” button on the computer screen to send their answers to their partner and click “RECEIVE” button to receive their partner’s responses, which included the partner’s evaluation form.

We used a similar evaluation form used in Mendes et al. (2008) to provide participants either positive or negative feedback. Specifically, the evaluation form listed the following five statements with the partner’s ostensible rating on each statement made on a scale of –4 to +4: “I would like to work at the same business or job as my partner,” “I would like to work closely on a project or team with my partner,” “I would like to get to know my partner better,” “I would enjoy being neighbors with my partner,” and “I would like to be close friends with my partner.” Participants in the positive feedback condition received favorable ratings on all five items (+3 for the first two statements and +4 for the rest three), while those in the negative feedback condition received generally unfavorable ratings (0 for the first three statements and –1, and –2 for the fourth, and fifth, respectively). We developed this slightly modified version because the feedback used in Mendes et al. (2008) targeted college students (e.g., “I would enjoy being roommates with the other subject”), whereas our participants were older and typically not college students.

Both the experimenters and confederates were kept unaware of the feedback manipulation; they were not only unaware to the type of the feedback but also to the fact that we were manipulating feedback at all. The authors were the only lab personnel who knew that this study included the feedback manipulation. These efforts to keep the manipulation a secret to our research staff and confederates protected against the possibility that the confederates, either consciously or unconsciously, attempted to modify their behavior to either align with or counter the presumed feedback. After participants reviewed the evaluation form, the experimenter returned to the room and asked participants to complete the *pre-interaction questionnaire*, which included measures of participants’ affective states as well as their partner perception.

#### Phase 5: In-person interaction

After the completion of the questionnaire, the experimenter moved the confederate to the participant’s room so that they could perform two interactive tasks together. They were told that depending on the joint performance on these two tasks, they could each earn an additional monetary bonus ($11). The participant and the confederate first engaged in a cooperative task, based on the game of taboo, where each player alternated providing clues for target words for 2 min without using any of the five “taboo” words listed on their prompt cards (see West et al., 2017). The dyad was told that they would receive points for every correct response and lose points if a taboo word was used. This task lasted for 8 min. The confederate’s performance and reactions were scripted during this task; it was pre-determined whether they would correctly guess or not during their turns as well as the prompts they provided to their partner during the participant’s turns. In addition, the confederates were trained to act in the same neutral way toward participants, regardless of how the participant acted toward them. After the dyad completed the game, participants filled out the *within-interaction questionnaire*, which included measures of affective states and anger expression.

The dyad then performed another interactive task (i.e., a tactile finger-spelling task; West et al., 2017) for 3 min.^2^ After the completion of this task, the confederate was moved back to their original room, and the participant was asked to complete the *post-interaction questionnaire* alone to assess their affective states and partner perception one more time. The participant then provided the second (T2) and third (T3) saliva samples, 18 and 33 min following the beginning of the taboo game, respectively.

#### Phase 6: Public goods provision task

After the third saliva assessment, the experimenter removed the physiological sensors and provided instructions for the public goods provision task. Participants were told that they and their partner each earned a total $11 bonus from the two interactive tasks they performed together and would both be asked to decide how much of the $11 they want to put in a “common pot.” They were told that the total money in the common pot would be multiplied by 1.5 point and divided equally between them (resulting in a maximum bonus of $16.5 for each). We used the amount of money participants put in the common pot as a behavioral index of cooperation (e.g., Ishii and Kurzban, 2008; *M* = 4.78 dollars, *SD* = 1.43). At the end of the task, we probed for suspicion and debriefed participants. All participants received the maximum $17 (rounded-up) bonus in addition to the $50 compensation.

### Measures

#### Cardiovascular responses

We obtained cardiovascular responses from participants with the intent to differentiate *challenge* and *threat* reactivity, which typically includes pre-ejection period (PEP; a measure of sympathetic nervous system [SNS] activation), cardiac output (CO; a measure of cardiac efficiency), and total peripheral resistance (TPR; a measure of overall vasoconstriction and vasodilation in the arterioles). To obtain these measures, we used impedance cardiography, electrocardiography, and blood pressure monitored throughout the study. Impedance cardiography was obtained with a HIC-2000 Bio-Electric Impedance Cardiograph (Bio-Impedance Technology, Chapel Hill, NC, United States), using a tetrapolar aluminum/mylar tape electrode system, which provided basal transthoracic impedance (Z0) and the first derivative basal impedance (dZ/dt). Electrocardiography was recorded with two Ag/AgCI electrodes placed in a modified Lead II configuration (right upper chest, left lower rib). These signals were interfaced with a Biopac MP150 data acquisition system (Goleta, CA, United States). All data were edited and scored off-line in 1-min bins using IMP (3.0) module from Mindware Technologies (Gahanna, OH, United States). We extracted PEP, CO, and heart rate (HR) as the primary measures of interest.

We also obtained continuous blood pressure responses to estimate TPR. Unfortunately, the blood pressure monitor we used (Continuous Non-invasive Arterial Pressure monitor: CNAP Monitor 500; CNSystems Medizintechnik AG, Grax, Austria) provided highly unstable and unreliable blood pressure responses from implausible values of 30 mmHg to 210 mmHg and this was exacerbated during the tasks likely due to participant movement that we could not control. Due to the invalidity of the blood pressure responses, we were thus unable to estimate TPR, which requires blood pressure responses.

Based on the available data we collected, we computed reactivity indices on three physiological parameters (i.e., HR, PEP, and CO). To examine how oxytocin, social feedback, and the interaction between the two influenced cardiovascular reactivity following the feedback, we computed change scores by subtracting participants’ baseline responses obtained during the last minute of the initial resting period from the physiological responses obtained during the first segment of the interactive task (i.e., taboo game) to yield each reactivity index (see Mendes et al., 2008 for a similar approach).^3^

#### Testosterone responses

Immediately following the experiment, the saliva samples were frozen at –80°C. Upon completion of the study, the samples were shipped on dry ice to Dirk Hellhammer’s lab at the University of Trier, Germany, where they thawed and spun at 3,000 rpms before assaying. The samples were analyzed for testosterone concentrations with an enzyme immunoassay kit (Salimetrics, State College, PA, United States). The lower limits of detection for testosterone were 1 pg/mL. The samples were assayed twice and the intra-assay coefficients of variation (CV) were 5.5, 5.5, and 6.3 for T1, T2, and T3 testosterone, respectively. The averaged data of the two assays were used for the analysis. We did not include low or high control samples in the assay plates to calculate inter-assay CVs. To adjust for gender difference in testosterone responses (e.g., Archer, 2006), we used scores standardized within gender in the analysis (see Maner et al., 2008 for a similar approach).^4^

#### Self-report measures

##### Partner perception

We assessed participants’ perception about their partner in two ways, based on their own liking toward their partner (i.e., how much I like my partner) and based on their inferred liking by the partner (i.e., how much I think my partner likes me). These assessments were obtained at two time points following the feedback manipulation—(a) immediately after reviewing the evaluation form but before the in-person interaction with the partner and (b) after the in-person interaction. First, participants’ partner liking before the in-person interaction was assessed with four items (e.g., “I am looking forward to meeting this person,” “This person is the type of person who would be my friend”; α = 0.86), on a 7-point scale (1 = *strongly disagree*, 7 = *strongly agree*). After the in-person interaction, participants rated their liking toward their partner again, based on five items (e.g., “I like my partner,” “I trust my partner”; α = 0.88). Second, participants’ inferred liking by the partner was assessed before the in-person interaction with two items (i.e., “My partner is looking forward to meeting me,” “My partner will like me”; α = 0.82). After the in-person interaction, participants once again rated their inferred liking based on four items (e.g., “My partner likes me,” “My partner trusts me”; α = 0.90).

##### Affective states

We measured participants’ global positive affect and negative affect with the Positive and Negative Affect Schedule (PANAS; Watson et al., 1988) at four time points throughout the study−(a) at baseline, (b) before their in-person interaction with the partner, (c) within the in-person interaction (i.e., after completing the first interactive task), and (d) after the in-person interaction. This allowed us to examine whether oxytocin modulates natural fluctuations in affective reactions over time after receiving the feedback, while controlling for baseline affect. At each time point, participants rated on a 5-point scale (1 = *not at all*, 5 = *a great deal*) the extent to which they felt 10 positive emotions (e.g., excited, active; αs ranged from 0.88 to 0.90) and 12 negative emotions (e.g., upset, hostile; αs ranged from 0.77 to 0.85).

##### Anger expression

After completing the taboo game, we assessed participants’ anger expression with the 24-item Anger Expression Scale (AX; Spielberger et al., 1986). Participants used a 4-point scale (1 = *strongly disagree*, 4 = *strongly agree*) to indicate the extent to which they felt like right now, outwardly expressing anger (anger-out; e.g., “slamming doors,” “saying nasty things”; α = 0.67), suppressing anger/hostility (anger-in; e.g., “I want to pout or sulk,” “I am boiling inside, but I am not showing it”; α = 0.73), and controlling anger expression (anger-control; e.g., “I control my angry feelings,” “I can stop myself from losing my temper”; α = 0.68).^5^

## Results

### Data analyses overview

During debriefing, three participants (one in oxytocin/negative feedback condition, one in oxytocin/positive feedback condition, and one in placebo/positive feedback condition) indicated that they were suspicious about the authenticity of their “partner” and believed that their partner was a confederate. Thus, the data from these participants were excluded from all analyses, which left 103 participants with analyzable data (58 women; *M*_age_ = 25.40, *SD*_age_ = 4.86).

Before data analyses, we checked outliers (i.e., responses outside the three interquartile range) in physiological responses and found three such values (one in PEP and two in CO). These values were retained in the analysis after being winsorized at the 90*^th^* percentile to minimize their impact (Jose and Winkler, 2008; Wilcox, 2011). Preliminary analyses showed that gender did not influence any of the outcome variables we assessed (except, not surprisingly, for testosterone, which we analyzed following typical analytic strategies based on gender differences in testosterone levels) and it also did not interact with intranasal spray and/or feedback to predict any of these variables, so we do not discuss this variable further. See Table 1 for descriptive statistics of key study variables (and see Supplementary Table 1 for inter-correlations).^6^

**TABLE 1:** Descriptive statistics of study variables assessed at four time points throughout the study.

Variables	Baseline		Before interaction		During interaction		After interaction	
	*M*	*SD*	*M*	*SD*	*M*	*SD*	*M*	*SD*
**Primary measures**								
Heart rate	67.34	9.14			83.92	13.08		
Pre-ejection period	118.05	11.41			107.97	11.89		
Cardiac output	5.81	2.35			6.21	2.62		
Cooperative behavior ($)							4.77	1.43
Partner liking			4.60	1.24			5.42	1.03
Inferred liking by partner			4.34	1.52			4.93	1.21
Positive affect	3.14	0.90	3.13	0.92	3.44	0.87	3.40	0.92
Negative affect	1.40	0.43	1.44	0.46	1.29	0.43	1.23	0.39
Anger expression					1.31	0.32		
Anger suppression					1.60	0.35		
Anger control					3.17	0.57		
**Testosterone (pg/mL)**								
Total sample	93.03	57.56			86.68	56.89	80.10	53.68
Males	139.88	54.80			132.90	54.03	125.00	48.81
Females	57.48	25.03			51.61	25.11	46.03	23.91
**Exploratory measures**								
Demand/resource appraisals					80.00	22.58		
Social touch (seconds)					0.57	0.31		
**Cortisol (ug/dL)**								
Total sample	4.83	3.54			3.71	2.43	3.39	2.25
Males	5.30	4.39			4.22	2.44	3.80	2.34
Females	4.48	2.71			3.32	2.37	3.08	2.15

Cardiovascular responses, cooperative behavior (the amount of money participants put in the common pot during the public goods provision task), and testosterone responses are the raw data before transformation. The results from the three exploratory measures are reported either as a footnote (see Footnote 4 for cortisol reactivity) or in the Supplementary Materials (for demand/resource appraisals and social touch, operationalized as the amount of time the dyad touched their hands during the tactile finger-spelling task). The second and third saliva samples were obtained at 18 and 33 min following the beginning of the in-person interaction, respectively.

We hypothesized that Black participants would respond more favorably following positive feedback from the White partner after the intranasal spray of oxytocin (vs. placebo), resulting in greater approach motivation indexed by challenge (vs. threat) patterns of cardiovascular reactivity, greater cooperative behavior, more favorable perceptions about their partner, and increased positive (vs. negative) affect. In contrast, we hypothesized that oxytocin would amplify negative emotional reactions following negative feedback, indexed by greater self-reported anger display and elevated testosterone reactivity.

To test our primary hypothesis, we conducted a 2 Intranasal spray (oxytocin vs. placebo) × 2 Feedback (positive vs. negative) analysis of variance (ANOVA) for each outcome variable. In addition, for certain outcome variables that were assessed more than one time point throughout the study, such as partner perception ratings (two times), affective states (four times, with the baseline value as a covariate), and testosterone reactivity (three times, with the baseline value as a covariate), we added a within-participants time factor to examine whether oxytocin effects manifest differently as a function of time following the feedback manipulation (i.e., Intranasal spray × Feedback × Time). We predicted that it may take time for oxytocin to exert its effects, such that the hypothesized effects might be stronger during or after the in-person interaction with the partner, rather than immediately following the feedback manipulation (but before the in-person interaction). For any significant interaction effect, we tested subsequent simple effects by applying Bonferroni corrections for multiple comparisons. Finally, as an exploratory analysis, we examined whether and how testosterone responses were associated with participants’ self-reported anger responses, to begin to address how these two different proxies of angry reactions might be related. See Table 2 for main results from all outcome variables.

**TABLE 2:** Summary of the results from the primary analysis (Intranasal spray × Feedback) and exploratory analysis (Intranasal spray × Feedback × Time).

		*F*-tests						
Variable	*N*	Intranasal spray	Feedback	Time	I × F	I × T	F × T	I × F × T
**Challenge vs. threat**								
CO reactivity	100	0.26	1.86		3.46^†^			
PEP reactivity	99	0.16	0.85		0.02			
Cooperative behavior	102	0.39	3.92*		5.87*			
**Partner perception**								
Partner liking	100	0.03	89.11***	127.94***	0.54	2.94^†^	14.28***	1.79
Inferred liking by partner	100	0.38	126.59***	25.01***	0.07	2.50	9.06**	3.96*
**Affective states**								
Positive affect	84	1.67	7.32***	6.95***	0.68	0.13	7.35***	0.09
Negative affect	84	0.54	5.75*	0.98	1.42	0.41	0.84	0.24
**Anger reactions**								
Anger expression	102	1.79	0.32	1.44				
Anger suppression	102	3.38^†^	3.56^†^	4.00*				
Anger control	102	1.12	1.13	0.02				
Testosterone reactivity	102	1.40	0.41	0.00	1.29	0.30	0.15	0.48

I × F, Intranasal spray × Feedback; I × T, Intranasal spray × Time; F × T, Feedback × Time; I × F × T, Intranasal spray × Feedback × Time. The analyses for affective states and testosterone reactivity were conducted controlling for their baseline values.

^†^*p* < 0.10, *p < 0.05, **p < 0.001, ***p < 0.001.

### Cardiovascular responses

First, we examined whether intranasal spray, feedback, and/or the interaction between the two influenced cardiovascular responses. All analyses focused on cardiovascular “reactivity” scores, computed by subtracting participants’ baseline responses from the responses obtained during the first 2 min of the cooperative task.

Before conducting our main analyses, we first examined if our paradigm successfully induced SNS activation among our sample (i.e., a necessary condition to differentiate challenge vs. threat reactivity; Blascovich and Mendes, 2000) by performing a 2 Intranasal spray × 2 Feedback ANOVA on HR and PEP reactivity scores, separately. Previous studies suggest that emotional responses following negative feedback such as anger can increase SNS activation more so than emotional responses following positive feedback such as experiences of high arousal positive emotions (Stemmler, 1989; Mendes et al., 2008; Kreibig, 2010; but see also Mendes and Park, 2014 for the moderating effects of contexts). We observed a similar pattern, such that participants in the negative feedback condition tended to show descriptively greater SNS activation—characterized with a greater increase in HR (*M* = 18.22, *SE* = 1.52) and a greater decrease in PEP (*M* = –11.31, *SE* = 1.38) from baseline levels—than those in the positive feedback condition (HR: *M* = 14.96, *SE* = 1.54; PEP: *M* = –9.51, *SE* = 1.39), but these effects did not reach statistical significance, *F*(1,95) = 2.27, *p* = 0.135, and *F*(1,95) = 0.85, *p* = 0.359, respectively. Neither the main effect of intranasal spray nor its interaction with feedback was significant, *F*s < 0.74, *p*s > 0.392. Importantly though, participants in all four conditions showed a significant increase in SNS activation from baseline levels, indexed by an increase in HR, *t*s > 7.02, *p*s < 0.001, Cohen’s *d*s > 1.46, and a decrease in PEP, *t*s > |–4.55|, *p*s < 0.001, Cohen’s *d*s > 0.89, thereby meeting the necessary condition to further explore challenge vs. threat reactivity.

**FIGURE 2:**
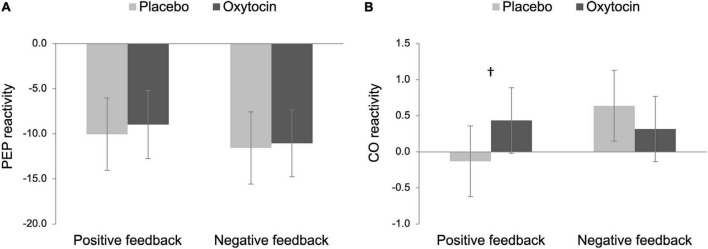
Pre-ejection period (PEP; **A**) and cardiac output (CO; **B**) reactivity as a function of intranasal spray (placebo vs. oxytocin) in each feedback condition. Lower scores on PEP reactivity indicate greater sympathetic nervous system (SNS) activation while higher scores on CO reactivity indicate greater cardiac efficiency. Challenge patterns of cardiovascular reactivity are characterized by an increase in SNS activity along with an increase in cardiac efficiency whereas threat patterns of cardiovascular reactivity are characterized by an increase in SNS along with no change in cardiac efficiency. Error bars indicate 95% confidence intervals. ^†^*p* < 0.10.

For our primary analysis, we then examined both PEP and CO reactivity to differentiate states of challenge vs. threat, following an established approach (Blascovich and Mendes, 2000; Mendes et al., 2008). Challenge states are characterized as an increase in SNS (a decrease in PEP) and cardiac efficiency (an increase in CO), whereas cardiovascular responses exhibited in threat states are associated with an increase in SNS and less efficient cardiac output (no change or a decrease in CO). As noted above and also shown in Figure 2A, participants in all four conditions showed a significant decrease in PEP from baseline, and thus, we examined CO reactivity to further differentiate states of challenge vs. threat.

The main effects of intranasal spray and feedback were not significant on CO reactivity, *F*s < 1.86, *p*s > 0.176. Importantly though, there was a trend of the interaction between intranasal spray and feedback, *F*(1,96) = 3.46, *p* = 0.066, η_p_^2^ = 0.04, 90% Confidence Interval (CI) [0.00, 0.11]. To decompose this interaction effect, we tested the simple effect of intranasal spray on CO reactivity in each feedback condition separately. In the positive feedback condition, the effect of intranasal spray approached statistical significance, *F*(1,96) = 2.80, *p* = 0.097, η_p_^2^ = 0.03, 90% CI [0.00, 0.10]; participants who were given oxytocin tended to show greater CO reactivity (*M* = 0.44, *SE* = 0.17), compared to those who were given placebo (*M* = −0.13, *SE* = 0.18) (see Figure 2B). Combined with a significant decrease in PEP, the pattern displayed by participants given oxytocin is consistent with a challenge-pattern of cardiovascular reactivity. In contrast, those given placebo showed a threat-pattern of cardiovascular reactivity, characterized as a smaller or no increase in CO combined with a decrease in PEP. Among those who received negative feedback, participants who were given oxytocin did not differ from those who were given placebo, *F*(1,96) = 0.92, *p* = 0.341. Both groups showed a challenge/approach-oriented pattern of reactivity (consistent with anger).

Taken together, we replicated Mendes et al. (2008) in the placebo conditions, such that Black participants exhibited a threat-pattern of cardiovascular reactivity in the positive feedback condition while exhibiting a challenge-pattern of cardiovascular reactivity in the negative feedback condition. Notably, oxytocin tended to reduce Black participants’ threat responses following outgroup partner’s positive feedback, such that only participants in the placebo/positive feedback condition exhibited threat responses whereas the other three groups all showed challenge/approach-oriented patterns. To formally test this group difference, we conducted a *post hoc* contrast analysis on CO reactivity to compare the placebo/positive feedback condition (–3) with the rest of the three conditions (all + 1 s). This analysis yielded a significant result, *F*(1,96) = 4.41, *p* = 0.038, η_p_^2^ = 0.04, 90% CI [0.00, 0.13], indicating that placebo participants showed greater threat responses following positive feedback whereas participants in the other three conditions showed challenge reactivity.

### Public goods provision

**FIGURE 3:**
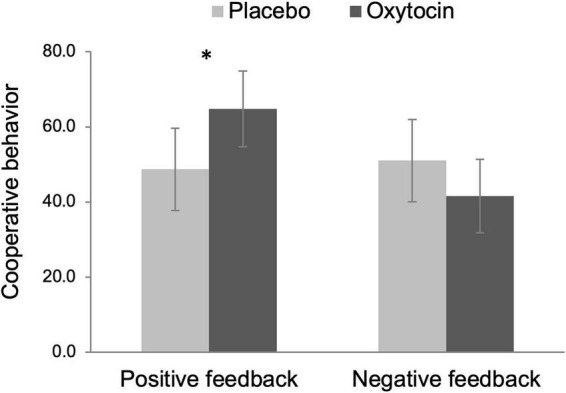
Cooperative behavior (i.e., the amount of money participants put in the common pot during the public goods provision task) as a function of intranasal spray (placebo vs. oxytocin) in each feedback condition. The data were rank-transformed to reduce skewness. Error bars indicate 95% confidence intervals. *p < 0.05.

We operationalized cooperation as the amount of money participants put in the common pot during the public goods provision task. Because this variable did not follow a normal distribution [*D*(102) = 0.35, *p* < 0.001, Kolmogorov–Smirnov test], we rank-transformed this variable before submitting it to a 2 Intranasal spray × 2 Feedback ANOVA (see Conover and Iman, 1981 for this recommended approach). The main effect of intranasal spray was not significant, *F*(1,98) = 0.39, *p* = 0.534, but there was a tendency that participants exhibited more cooperative behavior after receiving positive (vs. negative) feedback, *F*(1,98) = 3.92, *p* = 0.050, η_p_^2^ = 0.04, 90% CI [0.00, 0.12]. Importantly, there was a significant Intranasal spray × Feedback interaction effect, *F*(1,98) = 5.87, *p* = 0.017, η_p_^2^ = 0.06, 90% CI [0.01, 0.14]. As Figure 3 displays, among participants who received positive feedback, those given oxytocin exhibited greater cooperative behavior (*M* = 64.80, *SE* = 5.09) than those given placebo (*M* = 48.76, *SE* = 5.52), *F*(1,98) = 4.57, *p* = 0.035, η_p_^2^ = 0.05, 90% CI [0.00, 0.13]. In contrast, oxytocin did not modulate cooperative behavior after negative feedback, *F*(1,98) = 1.64, *p* = 0.203.

### Partner perceptions

Next, we analyzed partner perception ratings that participants completed prior to and immediately following the in-person interaction, based on (a) their own liking toward their partner (i.e., how much I like my partner) and (b) their inferred liking by the partner (i.e., how much I think my partner likes me).

First, participants’ partner liking ratings were submitted to a 2 Intranasal spray × 2 Feedback × 2 Time (before vs. after the in-person interaction) mixed ANOVA with intranasal spray and feedback as between-participant factors and time as a within-participant factor. This analysis yielded a significant main effect of feedback, *F*(1,96) = 89.11, *p* < 0.001, η_p_^2^ = 0.48, 90% CI [0.36, 0.57]. Consistent with a manipulation check, participants who received positive feedback liked their partner more (*M* = 5.78, *SE* = 0.11), compared to those who received negative feedback (*M* = 4.30, *SE* = 0.11). This finding is especially interesting because the confederates always acted with the same neutral affect toward participants and, indeed, were not aware that there was a feedback manipulation. In addition, the main effect of time was also significant, *F*(1,96) = 127.94, *p* < 0.001, η_p_^2^ = 0.57, 90% CI [0.46, 0.65]; in general, participants liked their partner more after the in-person interaction (*M* = 5.42, *SE* = 0.08), compared to before the in-person interaction (*M* = 4.66, *SE* = 0.09). These effects were qualified by a significant Feedback × Time two-way interaction effect, *F*(1,96) = 14.28, *p* < 0.001, η_p_^2^ = 0.13, 90% CI [0.04, 0.23], such that the effect of time was larger in the negative feedback condition, *F*(1,96) = 112.75, *p* < 0.001, η_p_^2^ = 0.54, 90% CI [0.43, 0.62], than in the positive feedback condition, *F*(1,96) = 28.65, *p* < 0.001, η_p_^2^ = 0.23, 90% CI [0.12, 0.34]. That is, participants who received negative feedback showed a greater increase in partner liking over time (before the interaction: *M* = 3.80, *SE* = 0.12; after the interaction: *M* = 4.81, *SE* = 0.12), compared to those who received positive feedback (before the interaction: *M* = 5.53, *SE* = 0.12; after the interaction: *M* = 6.03, *SE* = 0.12). In addition, there was a trend of the interaction between intranasal spray and time, *F*(1,96) = 2.94, *p* = 0.089, η_p_^2^ = 0.03, 90% CI [0.00, 0.10]; the effect of time tended to be larger among those who were given oxytocin, *F*(1,96) = 96.46, *p* < 0.001, η_p_^2^ = 0.50, 90% CI [0.38, 0.59], compared to those who were given placebo, *F*(1,96) = 41.09, *p* < 0.001, η_p_^2^ = 0.30, 90% CI [0.18, 0.41]. However, the critical two-way interaction between intranasal spray and feedback was not statistically significant, *F*(1,96) = 0.54, *p* = 0.464. There was also no evidence of a three-way interaction among intranasal spray, feedback, and time, *F*(1,96) = 1.79, *p* = 0.184.

Second, the same mixed ANOVA was performed on participants’ inferred liking by the partner. As similarly shown above, both main effects of feedback and time were significant, *F*(1,96) = 126.59, *p* < 0.001, η_p_^2^ = 0.57, 90% CI [0.46, 0.65] and *F*(1,96) = 25.01, *p* < 0.001, η_p_^2^ = 0.21, 90% CI [0.10, 0.32], respectively. Participants perceived their partner to have more favorable attitudes toward them after receiving positive feedback (*M* = 5.60, *SE* = 0.12), compared to negative feedback (*M* = 3.73, *SE* = 0.12), and after the in-person interaction (*M* = 4.92, *SE* = 0.09), compared to before the in-person interaction (*M* = 4.41, *SE* = 0.11). The effect of time, however, was only significant in the negative feedback condition (before the interaction: *M* = 3.31, *SE* = 0.15; after the interaction: *M* = 4.14, *SE* = 0.13), *F*(1,96) = 31.78, *p* < 0.001, η_p_^2^ = 0.25, 90% CI [0.13, 0.36], but not in the positive feedback condition (before the interaction: *M* = 5.50, *SE* = 0.15; after the interaction: *M* = 5.70, *SE* = 0.13), *F*(1,96) = 2.00, *p* = 0.160, resulting in a significant Feedback × Time two-way interaction effect, *F*(1,96) = 9.06, *p* = 0.003, η_p_^2^ = 0.09, 90% CI [0.02, 0.18]. Neither the Intranasal spray × Time interaction nor the Intranasal spray × Feedback interaction was significant, *F*(1,96) = 2.50, *p* = 0.117 and *F*(1,96) = 0.07, *p* = 0.786, respectively, but importantly, we found a significant Intranasal spray × Feedback × Time three-way interaction effect, *F*(1,96) = 3.96, *p* = 0.049, η_p_^2^ = 0.04, 90% CI [0.00, 0.12].

**FIGURE 4:**
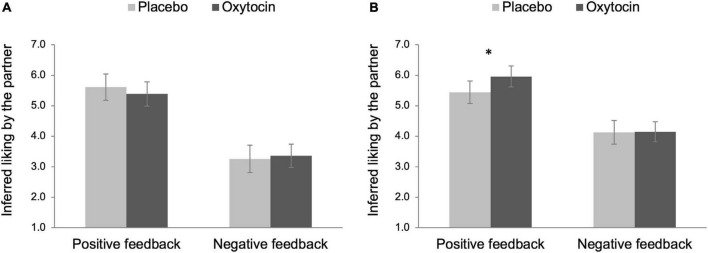
Partner perception before **(A)** and after **(B)** the in-person interaction as a function of intranasal spray (placebo vs. oxytocin) in each feedback condition. Higher number indicates that participants perceived their partner to have more favorable attitudes toward them. Error bars indicate 95% confidence intervals. *p < 0.05.

We decomposed the three-way interaction effect by testing simple effects of intranasal spray on participants’ inferred liking before and after the in-person interaction in each feedback condition separately. As shown in Figure 4A, there was no effect of intranasal spray on partner’s inferred liking before the in-person interaction for both feedback conditions, *F*s < 0.55, *p*s > 0.458. Intranasal spray also did not modulate partner’s inferred liking after the in-person interaction in the negative feedback condition, *F*(1,96) < 0.01, *p* = 0.952. In contrast, there was a significant effect of intranasal spray in the positive feedback condition, *F*(1,96) = 4.11, *p* = 0.046, η_p_^2^ = 0.04, 90% CI [0.00, 0.12], indicating that after receiving positive feedback, participants given oxytocin (*M* = 5.96, *SE* = 0.17) perceived their partner to have more favorable attitudes toward them after the in-person interaction, compared to those given placebo (*M* = 5.45, *SE* = 0.19) (see Figure 4B).

### Affective states

Next, we examined whether and how oxytocin modulated fluctuations in affective responses over time after receiving the feedback. We performed a 2 Intranasal spray × 2 Feedback × 3 Time (before the in-person interaction vs. during the interaction [i.e., after completing the first interactive task] vs. after the interaction) mixed ANCOVA separately for positive affect and negative affect, with intranasal spray and feedback as between-participant factors and time as a within-participant factor, while controlling for its baseline value.

When we examined participants’ positive affect, there were significant main effects of time and feedback, *F*(2,158) = 6.95, *p* = 0.001, η_p_^2^ = 0.08, 90% CI [0.02, 0.15] and *F*(1,79) = 7.32, *p* = 0.008, η_p_^2^ = 0.09, 90% CI [0.01, 0.19], respectively. In general, participants experienced greater positive affect both during and after the in-person interaction (*M* = 3.49, *SE* = 0.06 and *M* = 3.43, *SE* = 0.06, respectively) than before the in-person interaction (*M* = 3.17, *SE* = 0.05). Participants also reported higher levels of positive affect after receiving positive feedback (*M* = 3.50, *SE* = 0.07), compared to negative feedback (*M* = 3.23, *SE* = 0.07). These effects were qualified by a Feedback × Time interaction effect, *F*(2,158) = 7.35, *p* = 0.001, η_p_^2^ = 0.09, 90% CI [0.02, 0.15]; such that participants reported greater positive affect after receiving positive (vs. negative) feedback, especially before the in-person interaction (positive feedback: *M* = 3.43, *SE* = 0.07; negative feedback: *M* = 2.92, *SE* = 0.08), *F*(1,79) = 22.59, *p* < 0.001, η_p_^2^ = 0.22, 90% CI [0.10, 0.34], compared to during (positive feedback: *M* = 3.60, *SE* = 0.09; negative feedback: *M* = 3.38, *SE* = 0.09) or after the in-person interaction (positive feedback: *M* = 3.49, *SE* = 0.09; negative feedback: *M* = 3.38, *SE* = 0.09), *F*(1,79) = 3.14, *p* = 0.080, η_p_^2^ = 0.04, 90% CI [0.00, 0.13] and *F*(1,79) = 0.74, *p* = 0.391, respectively. No other effects, including the critical Intranasal spray × Feedback interaction, reached statistical significance, *F*s < 1.67, *p*s > 0.200.

Next, we performed the same mixed ANOVA on negative affect and found a significant main effect of feedback, *F*(1,79) = 5.75, *p* = 0.019, η_p_^2^ = 0.07, 90% CI [0.01, 0.17], indicating that participants reported greater negative affect after receiving negative feedback (*M* = 1.40, *SE* = 0.05) than positive feedback (*M* = 1.25, *SE* = 0.04). No other effects were statistically significant, *F*s < 1.42, *p*s > 0.237.

### Anger expression

We hypothesized that oxytocin would amplify negative emotional reactions following negative feedback, such as anger and aggression. To test this hypothesis, we first examined participants’ state levels of anger expression during the in-person interaction by submitting each subscale of AX to a 2 Intranasal spray × 2 Feedback ANOVA. The effects of intranasal spray and/or feedback were negligible on both anger expression, *F*s < 1.79, *p*s > 0.184, and anger control, *F*s < 1.13, *p*s > 0.291. However, there emerged a significant Intranasal spray x Feedback interaction on anger suppression, *F*(1,98) = 4.00, *p* = 0.048, η_p_^2^ = 0.04, 90% CI [0.00, 0.12]. In response to negative feedback, participants given oxytocin reported suppressing their anger less (*M* = 1.54, *SE* = 0.06) than placebo participants (*M* = 1.80, *SE* = 0.07), *F*(1,98) = 7.48, *p* = 0.007, η_p_^2^ = 0.07, 90% CI [0.01, 0.16]. In contrast, oxytocin did not influence participants’ tendency to suppress anger in response to positive feedback, *F*(1,98) = 0.01, *p* = 0.909.

### Testosterone reactivity

Finally, we analyzed gender-adjusted testosterone responses obtained both at T2 and T3 (18 and 33 min following the beginning of the in-person interaction) by controlling for participants’ baseline (T1) testosterone responses to examine whether oxytocin intensified experience of anger and dominance following social rejection, possibly indexed by elevated testosterone reactivity. Specifically, we performed a 2 Intranasal spray × 2 Feedback × 2 Time (T2 vs. T3) mixed ANCOVA, while controlling for T1 testosterone responses. Neither the main effects nor the interactions between the predictor variables were statistically significant, *F*s < 1.40, *p*s > 0.240.

These results suggest that there was no evidence that intranasal oxytocin modulated testosterone reactivity differently as a function of feedback type at the group level. Nonetheless, we sought to examine whether self-reported anger responses predicted testosterone reactivity in each feedback condition, to begin to address how these two different proxies of anger reactions might be related at the individual level. We tested these associations separately for T2 and T3 testosterone reactivity after combining both spray conditions, as there was no effect of intranasal oxytocin on both variables. First, we regressed T2 testosterone levels on three subscales of anger expression as well as T1 testosterone as a baseline in each feedback condition. There was no significant relationship between anger expression and T2 testosterone in the negative feedback condition, *b* = 0.71, 95% CI [–0.19, 1.61], *t*(47) = 1.29, *p* = 0.120. In addition, anger control tended to predict greater T2 testosterone, *b* = 0.38, 95% CI [–0.02, 0.77], *t*(47) = –1.91, *p* = 0.062. In contrast, we found a significant negative relationship between anger suppression and T2 testosterone, *b* = –0.75, 95% CI [–1.50, –0.01], *t*(47) = −2.03, *p* = 0.048, indicating that the less participants suppressed their anger during the in-person interaction following social rejection, the greater testosterone reactivity they showed after the interaction. None of the anger subscales predicted T2 testosterone reactivity in the positive feedback condition, *t*s < 0.89, *p*s > 0.377.

Second, we conducted the same analyses with T3 testosterone reactivity. As similarly shown at T2, anger suppression negatively predicted T3 testosterone in the negative feedback condition, *b* = –1.13, 95% CI [–1.95, –0.31], *t*(47) = –2.78, *p* = 0.008. In addition, anger expression also tended to predict greater T3 testosterone, *b* = 0.91, 95% CI [–0.08, 1.90], *t*(47) = 1.86, *p* = 0.070. However, anger control was not associated with testosterone reactivity at T3, *b* = 0.03, 95% CI [–0.40, 0.47], *t*(47) = 0.16, *p* = 0.874. None of these subscales were significantly associated with T3 testosterone in the positive feedback condition, *t*s < 0.94, *p*s > 0.353.

To summarize, one consistent pattern we found across both time points is that anger suppression was *negatively* associated with testosterone reactivity in the negative feedback condition, such that those who suppressed their anger less after receiving negative feedback from their partner showed elevated testosterone reactivity both at T2 and T3.^7^,^8^

### Robustness checks

We used the conventional threshold with a *p*-value of 0.05 to determine statistical significance for each outcome variable. However, given that we performed simultaneous hypothesis testing on multiple outcomes (a total of 11 univariate tests; see Table 2), this can inflate the probability of Type I error (i.e., erroneously rejecting the null hypothesis). Thus, as a robustness check, we applied the Benjamini–Hochberg (B-H) procedure to control for the false discovery rate (FDR) for multiple testing (Benjamini and Hochberg, 1995). To evaluate our results with different levels of stringency, we performed the B-H correction with FDR thresholds of 5, 10, and 20%, which indicate that roughly 5, 10, or 20% of all significant results are interpreted as possible false positives. Specifically, we rank-ordered the raw p-values of 11 outcome variables (from lowest to highest) and compared them with their B-H critical values calculated with the FDRs of 5, 10, and 20%, respectively. When the raw *p*-value is smaller than its B-H critical value, the hypothesis testing for this variable and all testing with *p*-values smaller than the *p*-value of this variable are considered statistically significant (McDonald, 2015). As summarized in Table 3, all significant results survived the correction with the FDR of 20%. Notably, the result on CO reactivity, which did not reach statistical significance in our original analysis (raw *p*-value = 0.066), proved to be statically significant with this correction. However, when we applied the correction with more stringent rates of 5 or 10%, none of the effects passed these additional tests.

**TABLE 3:** Descriptive statistics for robustness checks.

Outcome variable	Analysis	Raw *P*-value	i (rank)	B-H critical value (5% FDR)	B-H critical value (10% FDR)	B-H critical value (20% FDR)	Minimal detectable effect	Observed effect
Cooperative behavior	I × F	0.017	1	0.005	0.009	**0.018**	0.28	0.23
Anger suppression	I × F	0.048	2	0.009	0.018	**0.036**	0.28	0.19
Inferred liking by partner	I × F × T	0.049	3	0.014	0.027	**0.055**	0.17	0.20
CO reactivity	I × F	0.066	4	0.018	0.036	**0.073**	0.28	0.18
Partner liking	I × F × T	0.184	5	0.023	0.045	0.091	0.17	0.13
Anger expression	I × F	0.233	6	0.027	0.055	0.109	0.28	0.12
Testosterone	I × F × T	0.489	7	0.032	0.064	0.127	0.17	0.07
Negative affect	I × F × T	0.790	8	0.036	0.073	0.145	0.15	0.05
PEP reactivity	I × F	0.887	9	0.041	0.082	0.164	0.28	0.01
Anger control	I × F	0.898	10	0.045	0.091	0.182	0.28	0.01
Positive affect	I × F × T	0.911	11	0.050	0.100	0.200	0.15	0.03

I × F, Intranasal spray × Feedback; I × F × T, Intranasal spray × Feedback × Time. B-H critical value was computed using the following equation = (i/m) × Q, where *i* indicates the rank of the raw *p*-value, *m* indicates the total number of tests (11), and *Q* indicates the false discovery rate (FDR; 5, 10, or 20%) (Benjamini and Hochberg, 1995). The bolded numbers indicate the B-H critical values of four outcome variables that survived the FDR correction of 20%. Minimal detectable effect for each outcome was calculated based on a sensitivity power analysis while observed effect was based on an actual analysis (both indicate Cohen’s *f*).

As another way to check the robustness of our results, we conducted a sensitivity power analysis to identify a minimal detectable effect for each outcome variable. The sensitivity power analysis using G*Power (Faul et al., 2009) showed that our primary analysis (i.e., the Intranasal spray × Feedback interaction) based on our final sample (*N* = 103) was sufficient to detect a medium size effect with Cohen’s *f* = 0.28 (power = 0.80, α = 0.05, two-tailed). In addition, for our exploratory analysis that involved repeated measures (i.e., partner perceptions, affective states, and testosterone reactivity), we calculated a minimum detectable effect of the three-way interaction effect (Intranasal spray × Feedback × Time), assuming a correlation of 0.50 among the repeated measures. We expected that participants’ initial responses would serve as an anchor to affect their subsequent reactions, thereby yielding a medium-sized correlation among the repeated measures. The sensitivity power analyses showed that we had 0.80 power to detect the three-way interaction with a small size effect (Cohen’s *f* = 0.17 and 0.15 for partner perceptions and testosterone reactivity [with two levels] and affective states [with three levels], respectively). Next, we compared these minimal effect sizes with the observed effect sizes from our actual analyses. As shown in Table 3, the observed effect was smaller than the minimal detectable effect for all outcome variables, except for one variable; for inferred liking by partner, the observed effect size (Cohen’s *f* = 0.20) exceeded the minimal detectable effect calculated by the sensitivity power analysis (Cohen’s *f* = 0.17), suggesting that we had a sufficient power to detect the effect for this variable. However, our study was generally underpowered to observe the obtained effect for all other variables, and thus, caution is necessary to interpret these results.

## Discussion

What might seem to be a counter-intuitive finding—ethnic/racial minoritized individuals often react negatively to outgroup acceptance—is a common finding in intergroup literature that is predicted by attributional ambiguity theory. This theory proposes that minoritized individuals have additional attributional explanations for White partners’ positive behaviors; they perceive the positive behaviors driven by White individuals’ concerns over appearing prejudiced rather than reflecting genuine social acceptance. Theoretically, this explanation suggests that distrust is an important underlying mechanism. The key contribution of our work was to test this premise by examining the effects of intranasal oxytocin on Black participants’ physiological, affective, and behavioral responses to outgroup acceptance and rejection. Our results showed that oxytocin exerted divergent effects depending on the type of feedback Black participants received from the White partner.

### The role of oxytocin in positive interracial interaction

First, consistent with prior work documenting minoritized individuals’ negative reactions to positive feedback from White partners (Crocker et al., 1991; Hoyt et al., 2007; Mendes et al., 2008), we found that Black participants in the placebo condition reacted to positive feedback with cardiovascular reactivity characteristic of threat (i.e., less cardiac efficiency). In contrast, this deleterious reaction tended to be attenuated in the oxytocin condition. Instead, oxytocin facilitated more benign responses, including greater cardiac efficiency, greater cooperative behavior, and more favorable partner perceptions over time. Oxytocin did not increase participants’ own liking of their partner, but it enhanced participants’ inferred liking by their partner after (vs. before) the in-person interaction. This result is in line with a recent finding that oxytocin facilitates more favorable inferences about other people’s intentions, especially during positive social interactions (i.e., when these others were more generous during an economic decision-making game; Zhang et al., 2020). Taken together, our results suggest that oxytocin may have reduced Black participants’ suspicion stemming from attributional ambiguous treatments, which in turn, led them to react more favorably to the positive feedback based on more intuitive responses (i.e., my partner is nice to me, so I am nice to him/her). These results are also consistent with the finding that when social acceptance is perceived as genuine, this can yield equally positive effects on both minority and majority members (Kunstman et al., 2013).

### The role of oxytocin in negative interracial interaction

We had hypothesized that oxytocin might amplify angry reactions following outgroup rejection. We found mixed evidence for this hypothesis, depending on how anger responses were assessed. When self-reported anger responses were tested, Black participants given oxytocin (vs. placebo) reported suppressing their anger less during their interaction with the partner. This result is in support of our hypothesis and also consistent with prior findings that oxytocin facilitates angry reactions when the context involves negative interpersonal cues (Bosch et al., 2005; DeWall et al., 2014; Ne’eman et al., 2016). However, oxytocin did not modulate testosterone reactivity following negative feedback. Yet, our exploratory analysis showed a suggestive link between these two different proxies of anger reactions at the individual level. That is, the less participants suppressed anger during the interaction with their partner, the greater testosterone reactivity they displayed after the interaction. Oxytocin reduced Black participants’ regulatory efforts to modulate their angry feelings following social rejection, which in turn, may have gradually increased their anger experience following the interaction, possibly indexed by the elevated levels of testosterone reactivity (Mehta et al., 2008).

Our results may seem at odds with a recent finding that oxytocin enables people to cope with an experience of rejection better (Pfundmair and Echterhoff, 2021). Yet, one critical difference between their study and ours lies in the source of rejection. It is established from the intergroup literature that social rejection from an “outgroup” member engenders antagonistic reactions (Major et al., 2002; Mendes et al., 2008). We had hypothesized, guided by the social salience hypothesis, that oxytocin would amplify such reactions when the context involves negative interpersonal cues−that is, the presence of an outgroup member as a source of rejection. In contrast, the group status of the interaction partner was not made salient in Pfundmair and Echterhoff (2021) (i.e., using avatars in the Cyberball game), which may have attenuated the deleterious reactions typically following social rejection. Future research is necessary to test this speculation by directly manipulating the group status of the interaction partner.

### The social salience hypothesis

Taken together, our findings suggest that oxytocin exerts contrasting effects depending on the nature of social contexts. Some over-hyped early reports of oxytocin focused on the seemingly uniformly positive effects, but a large and growing literature identifies the critical contextual and individual differences that can moderate oxytocin effects (e.g., Shamay-Tsoory et al., 2009; Mikolajczak et al., 2010; Bartz, 2016). Building on this evidence, it has been proposed that oxytocin enhances perceptual salience of interpersonal cues, thereby yielding both positive and negative responses depending on the available social stimuli in a given context (Bartz et al., 2011; Olff et al., 2013; Shamay-Tsoory and Abu-Akel, 2016). Our results are consistent with this hypothesis by showing that oxytocin promotes different profiles of affective and social responses depending on the type of feedback people receive during interracial encounters. Furthermore, in support of the formulation that the enhanced salience of social cues, enabled by oxytocin, may motivate people to initiate intuitive and spontaneous actions instead of deliberate and calculated responses (Ma et al., 2015; Ten Velden et al., 2016), we found that participants who were given oxytocin, relative to placebo, exhibited more intuitive reactions to the feedback. In particular, the finding that participants given oxytocin (vs. placebo) responded more favorably to the positive feedback is consistent with emerging evidence that the prosocial effects of oxytocin are modulated by the amygdala-hippocampal circuitries—the brain regions that are recruited more for intuitive, affective processing than for deliberative, controlled processing (Baumgartner et al., 2008; De Dreu et al., 2015).

### Oxytocin and intergroup processes

Our work further extends the current literature on the role of oxytocin in intergroup contexts by showing that its prosocial effects are not confined to the boundary of ingroups. In a series of studies, De Dreu et al. (2010), De Dreu (2012; see also Sheng et al., 2013) found that the effects of oxytocin were moderated by group membership; oxytocin facilitated altruistic responses toward ingroup members while it increased defensive aggression and derogation against outgroup members. At first glance, these findings may seem at odds with our result that oxytocin promoted liking and cooperation toward *outgroup* members. We believe that the discrepancy between our finding and the De Dreu et al. (2010) is likely due to the fact that whereas we observed the effects of oxytocin under the condition where *positive* social cues were present (i.e., following positive social feedback), De Dreu et al. (2010) examined the role that oxytocin plays in intergroup competition, a context in which *negative* information about outgroups members was made salient. Consistent with this formulation, one study showed that oxytocin promotes prosocial behaviors toward outgroup members in the context of intergroup cooperation (Israel et al., 2012). Similarly, in the absence of salient negative cues, oxytocin increases empathetic reaction to outgroup members’ pain (Shamay-Tsoory et al., 2013). Thus, our results do not necessarily contradict the De Dreu et al. (2010), as both studies show that oxytocin increases sensitivity to the available social cues—that is, social feedback and group membership, respectively. To further examine the independent effects of these contextual factors, future research is needed to examine how individuals would respond to different types of social feedback provided either by a same-race or a different-race partner. Our study design did not allow us to address this issue given our focus on Black participants only. Future extensions of this work are necessary to examine whether the modulating effects of oxytocin documented in the current work are generalizable to different racial groups or uniquely observed among minority members.

### Statistical concerns

It is important to note two statistical concerns about our results. First, when we checked the robustness of our results by applying corrections for multiple testing, none of our significant results survived the B-H correction, especially when the stringent FDR thresholds of 5 or 10% were applied. Another related concern is that the *p*-values of most of our significant effects were just under the conventional threshold of 0.05 (i.e., *p*s = 0.048 and 0.049 for anger suppression and partner perception, respectively). Thus, when a more conservative threshold is used (e.g., *p* < 0.005, as recently proposed by Benjamin et al., 2018), none of our results may survive. In addition, our analysis on challenge/threat reactivity, especially the *post hoc* contrast analysis, was based on the data-driven approach, which could have inflated the risk of Type I error (Simmons et al., 2011; Button et al., 2013; Gelman and Loken, 2014). Taken together, these points suggest a possibility that our results may include false positives.

It is important to note, however, that the use of a stringent FDR threshold for multiple testing (e.g., 5%), while effective at lowering the probability of false positives, can obscure any effects that are actually present by increasing the risk of Type II error (i.e., failure to reject a false null hypothesis). The cost of missing a potentially important finding might be higher than the cost of false positives, especially during an initial discovery stage. Our study was the first investigation to test the role of intranasal oxytocin within interracial contexts among a community sample of Black Americans by combining multiple methods from social/personality psychology, psychophysiology, and psychoneuroendocrinology. Given our novel approach, a less stringent threshold might be more appropriate to identify potentially interesting findings during this initial discovery. Importantly, when we applied the less stringent threshold with the FDR of 20% to reduce the probability of Type II error, all our significant results remained significant.

The second concern is about statistical power. As another way to check robustness, we compared the minimal detectable effect calculated based on the sensitivity power analysis with the actual, observed effect for each outcome variable. The sensitivity power analyses showed that the current sample size (*N* = 103) would have been sufficient to detect a small-to-medium effect, and yet, the actually observed effect size was smaller for most variables (except for one variable), suggesting that our study was not sufficiently powered. This may be concerning because underpowered studies are more prone to producing Type I errors with inflated effect sizes (Ingre, 2013). Consistent with this view, in their recent review, Walum et al. (2016) concluded that intranasal oxytocin studies are typically underpowered, and thus, most published findings might actually be false positives. Another problem of underpowered studies is a failure to detect true effects that are actually present (i.e., false negatives). It is possible that the relatively low power of the current work made it more susceptible to Type II error, thereby resulting in some weak effects. A pre-registered replication with a larger sample is necessary to test our hypothesis more reliably.

### Limitations and future directions

Several limitations should be noted before concluding. First, to minimize the possible impact of the confederates, they were kept unaware of the feedback manipulation and were also trained to act in the same neutral way regardless of how the participant acted toward them. Yet, we were still not able to hold their reactions completely constant, and this may have added some variance to our results. Second, not having blood pressure responses prevented us from providing a complete replication of Mendes et al. (2008) and limits the certainty of claiming that the physiological responses are consistent with “threat” or “challenge” (see also Kubzansky et al., 2012 for a similar case related to invalid blood pressure readings). Changes in PEP and CO are consistently related to challenge and threat responses over the past 20 years of this work, and furthermore, CO responses are correlated with TPR (indeed, CO is part of the TPR equation = mean arterial blood pressure divided by CO), and yet, there is no question that not having TPR as one of the critical measures that differentiate challenge and threat is a limitation. Finally, there are several limitations regarding the testosterone measurement, such as missing information about inter-assay CVs. In addition, the use of immunoassays is less optimal than mass spectrometry-based measurement as it is known to overestimate testosterone levels among females, thereby reducing the actual gender difference in testosterone levels (Schultheiss et al., 2018). Future work should replicate and extend the current finding with the use of mass spectrometry measurement.

## Conclusion

The current research examined the role that intranasal oxytocin plays in influencing Black participants’ responses to outgroup acceptance and rejection. It provided the initial evidence in support of our thesis that oxytocin may enhance trust in positive interracial encounters, while amplifying negative reactions to outgroup rejection. It also highlights the need for future research to refine our knowledge concerning how oxytocin and social contexts jointly interact to influence intergroup interactions.

## Data availability statement

The datasets presented in this study can be found in online repositories. The names of the repository/repositories and accession number(s) can be found in the article.

## Ethics statement

The studies involving human participants were reviewed and approved by UCSF Committee on Human Research. The participants provided their written informed consent to participate in this study.

## Author contributions

WBM designed the study, oversaw scoring of physiologic data, and contributed to analyses and writing. JP supervised the study protocol and took the lead on analyzing the data and writing the manuscript. JW supervised the study protocol and contributed to data interpretation and manuscript writing. All authors contributed to the article and approved the submitted version.

## Footnotes

1 The current design allowed us to conceptually replicate Mendes et al. (2008), with the following three differences. First, Mendes et al. (2008) tested both White and Black participants, but in this study we tested Black participants only, as an attempt to examine the mechanism underlying minorities’ paradoxically negative reactions to positive feedback by White individuals, motivated by the attributional ambiguity theory. Second, several measures used by Mendes et al. (2008) were not included in the current work; (a) The self-report measure of attributions to discrimination was not administered because we were concerned that it would make race salient and contaminate other outcomes. (b) We did not analyze dyads’ performance during the cooperative task because the confederate’s performance was scripted during the task to minimize their possible impact. (c) Mendes et al. (2008) analyzed participants’ non-verbal behavior and emotional displays by coding the videotaped cooperative task that is not included in this project. Three, we administered several exploratory measures, including self-report measures of demand/resource appraisals, a tactile finger-spelling task, and saliva collection for hormone responses. We report results from most of these exploratory measures in the Supplementary Materials.

2 During this task, the confederate was instructed to spell out target words using the letters of American Sign Language (ASL), and the participant had to guess the words by touching their partner’s hand. The dyad each put their dominant hands inside a box placed on the table between them so that they could not see each other’s hand and had to feel their partner’s hand to guess the words. This task was designed to force the dyad to touch, which may be more uncomfortable for people who are less familiar with interracial encounters (see Supplementary Materials for more details).

3 The last minute of the resting period responses did not differ as a function of intranasal spray and/or feedback, except that there was a main effect of intranasal spray on the baseline PEP, *F*(1,96) = 6.20, *p* = 0.015, η_p_^2^ = 0.06, 90% CI [0.01, 0.15]. Participants who were given oxytocin exhibited lower levels of baseline PEP (*M* = 115.97, *SE* = 1.41) compared to those who were given placebo (*M* = 121.15, *SE* = 1.53). To adjust for this baseline difference, when we analyzed PEP reactivity by controlling for participants’ baseline PEP, this did not change the results. Similarly, instead of using the difference scores to yield each reactivity index, analyzing the physiological responses obtained during the first segment of the interactive task after controlling for the baseline responses did not substantially alter the results.

4 In addition to assaying testosterone, we also assayed cortisol. We did not have an *a priori* prediction about how oxytocin might influence stress hormones in each feedback condition, and yet, given that some prior research found the modulating effects of oxytocin on stress reactivity (Cardoso et al., 2014), we analyzed this variable for an exploratory purpose. As saliva samples were obtained at three time points−at baseline (T1) and at 18 min and 33 min following the onset of the first interactive task (T2 and T3, respectively), we performed a 2 Intranasal spray × 2 Feedback × 2 Time (T2 vs. T3) mixed ANCOVA, while controlling for T1 cortisol responses. We also controlled for gender to adjust for possible gender differences in cortisol reactivity (Kudielka and Kirschbaum, 2005). Neither the main effects nor the interactions between the predictor variables were statistically significant, *F*s < 2.29, *p*s > 0.133, suggesting that there was no evidence that oxytocin and/or feedback modulated stress reactivity.

5 The materials and data for the current paper are available at Open Science Framework (OSF): https://osf.io/xbfh4/?view_only=1daab5fc6856465db54afb165156bcf3.

6 Some studies suggest that oxytocin effects may depend on individual’s menstrual cycle phase (e.g., Engel et al., 2019). We thus examined whether the menstrual cycle phase (follicular [41.4%] vs. luteal [55.2%]) influenced the results among participants who menstruated. None of the outcome variables was predicted by this variable, *F*s < 1.42, *p*s > 0.239, except that those in the luteal phase shared more money during the public goods provision task than those in the follicular phase, *F*(1,53) = 4.46, *p* = 0.039, η_p_^2^ = 0.08, 90% CI [0.00, 0.20]. However, when we adjusted for the effect of the menstrual cycle, this did not alter the results from our main analysis. Specifically, we regressed the amount of shared money on the menstrual cycle phase and analyzed the residuals from this regression analysis for those who menstruated while analyzing the original data for those who didn’t menstruate. As shown in the main analysis, the Intranasal spray × Feedback interaction was statistically significant, *F*(1,96) = 4.15, *p* = 0.044, η_p_^2^ = 0.04, 90% CI [0.00, 0.12]. In addition, we also tested whether the use of hormonal contraceptives (yes [27.6%] vs. no [70.7%]) modulated our results. Several outcomes were predicted by this variable, including participants’ partner liking and positive affect before the in-person interaction and testosterone responses both at T2 and T3, *F*s > 3.35, *p*s < 0.073, η_p_^2^s < 0.06. However, when we controlled for this variable, this did not change the pattern of our results.

7 We used testosterone responses standardized within gender to adjust for gender differences in testosterone levels (e.g., Maner et al., 2008). As another way to address the gender differences, we also tested if gender moderated the relationships between anger suppression and testosterone reactivity both at T2 and T3, and it did not, *t*s < 0.91, *p*s > 0.370. Nonetheless, when we conducted the regression analyses separately for each gender group using the raw data before standardization, the relationship between anger suppression and testosterone reactivity was only significant among women at T3, *b* = −21.35, 95% CI [–40.33, –2.36], *t*(52) = –2.26, *p* = 0.028. The same pattern of the relationship was shown among men at T3 and also among both gender groups at T2, but none of them researched statistical significance, *t*s > ∣–1.06∣, *p*s < 0.293.

8 Caution is due in interpreting the results from these exploratory analyses, because when we formally tested the moderating effect of feedback (i.e., Feedback × Anger suppression interaction), the moderation was not significant at both time points, *t*s < 0.57, *p*s > 0.568. That is, there is no evidence that the relationship between anger suppression and testosterone reactivity is limited to the negative feedback condition.
