# Supplementary material for: The effects of intranasal oxytocin on black participants’ responses to outgroup acceptance and rejection
Source: Front Psychol. 2022 Aug 18;13:916305. doi: 10.3389/fpsyg.2022.916305 (PMC9434127; doi:10.3389/fpsyg.2022.916305)
Supplement: Supplementary file 1 [file Data_Sheet_1.docx]

Supplementary Materials for

**The Effects of Intranasal Oxytocin on Black Participants’ Responses to Outgroup Acceptance and Rejection**

Jiyoung Park^1^, Joshua Woolley^2^, & Wendy Berry Mendes^2^

^1^ Department of Psychology, University of Texas at Dallas

^2^ Department of Psychiatry, University of California, San Francisco

In this section, we present results from additional analyses we conducted on two exploratory measures; demand/resource appraisals and social touch.

**Demand/Resource Appraisals**

After participants were informed about how to play the taboo game, but before they started the task, participants were asked to complete a pre-task questionnaire designed to assess their challenge/threat states based on perceived demands and resources (Mendes et al., 2007). Although it is expected that demand/resource appraisals are associated with cardiovascular reactivity of challenge/threat, several studies documented the lack of convergence between the two, suggesting that challenge/threat states can be achieved without conscious awareness of the appraisal mechanisms (Blascovich et al., 2002; Park et al., 2021; Turner et al., 2012; Turner et al., 2014). We thus did not make a strong prediction regarding how oxytocin may interact with feedback to predict the demand/resource appraisals.

Five items assessed perceived demands (e.g., “I am very uncertain about how I will perform during the upcoming task,” “The upcoming task is very demanding”; α = .75, *M* = 2.79, *SD* = 1.11) and four items assessed perceived resources (e.g., “I have the abilities to perform the upcoming task successfully,” “It is very important to me that I perform well on this task”; α = .73, *M* = 5.24, *SD* = 1.03). The demand subscale was then divided by the resource subscale to create a threat (vs. challenge) ratio index, with higher scores indicating a greater threat (relative to challenge) (*M* = 0.57, *SD* = 0.31). We submitted this threat ratio index to a 2 Intranasal spray (oxytocin vs. placebo) x 2 Feedback (positive vs. negative) analysis of variance (ANOVA) and found a significant main effect of feedback, *F*(1, 96) = 4.77, *p* = .031, η_p_^2^ = 0.05, 90% CI [0.00, 0.13]. The threat index was higher for participants in the negative feedback condition (*M* = 0.64, *SE* = 0.04) than those in the positive feedback condition (*M* = 0.51, *SE* = 0.04). In addition, those given oxytocin tended to perceive less threat (vs. challenge) about the upcoming task (*M* = 0.53, *SE* = 0.04) than those given placebo (*M* = 0.63, *SE* = 0.04), although this effect of intranasal spray did not reach statistical significance, *F*(1, 96) = 2.94, *p* = .090, η_p_^2^ = 0.03, 90% CI [0.00, 0.10]. In addition, the interaction effect between intranasal spray and feedback was not statistically significant, *F*(1, 96) < 0.01, *p* = .993.

**Social Touch**

We also explored how intranasal spray and/or feedback influenced social comfort participants felt during the interaction with their partner by using a tactile finger-spelling task developed in our lab. After completing the first interactive task (i.e., taboo game), the dyad performed the tactile finger-spelling task. The confederate was instructed to spell out target words using the letters of American Sign Language (ASL), and the participant had to guess the words by touching their partner’s hand. The dyad each put their dominant hands inside a box placed on the table between them so that they could not see each other’s hand and had to feel their partner’s hand to guess the words. This task was designed to force the dyad to touch, which may be more uncomfortable for people who are less familiar with interracial encounters ([Olsson et al., 2005](#_ENREF_3)).

The amount of time the dyad touched their hands was assessed as a behavioral index of social comfort. Following an established protocol from previous studies ([Koslov, 2010](#_ENREF_1); [Koslov et al., 2014](#_ENREF_2); [Stern & West, 2014](#_ENREF_4)), trained research assistants independently scored the data based on the signal interference on the impedance waveforms caused by skin-to-skin touch to assess the amount of time the participant touched the confederate’s hand. The same data were scored by a second rater from the same group of the research assistants, to determine inter-rater reliability. We averaged the responses by two raters to create a single index of social comfort (α = .99, *M*_sec_ = 80.27, *SD*_sec_ = 22.44). As noted in the main document, we considered two opposing predictions. One might expect that oxytocin would enhance social touch in inter-racial interactions following positive feedback. On the other hand, we also reasoned that given the very intimate nature of social touch, the effect of oxytocin might not be sufficiently strong to enhance such an intimate, affiliative behavior, especially between strangers who do not have any relationship history. Consistent with the second possibility, when we entered the social touch index to a 2 Intranasal spray x 2 Feedback ANOVA, neither the main effects of intranasal spray and feedback nor the interaction between the two were significant, *F*s < 1.29, *p*s > .258.

**Supplementary References**

Blascovich, J., Mendes, W. B., & Seery, M. D. (2002). Intergroup threat: A multi-method approach. In D. M. Mackie & E. R. Smith (Eds.), *From prejudice to intergroup emotions: Differentiated reactions to social groups* (pp. 89-109). Psychology Press.

Koslov, K. (2010). *Pounding hearts and brittle smiles: The effect of resource depletion on outgroup positivity*[Dissertation]. Harvard University.

Koslov, K., Page-Gould, E., & Mendes, W. B. (2014). *Affect contagion during inter-racial interactions* Harvard University.

Mendes, W. B., Gray, H. M., Mendoza-Denton, R., Major, B., & Epel, E. S. (2007). Why egalitarianism might be good for your health: physiological thriving during stressful intergroup encounters. *Psychological Science*, *18*(11), 991-998. <https://doi.org/10.1111/j.1467-9280.2007.02014.x>

Olsson, A., Ebert, J. P., Banaji, M. R., & Phelps, E. A. (2005). The role of social groups in the persistence of learned fear. *Science*, *309*(5735), 785-787. <https://doi.org/10.1126/science.1113551>

Park, J., Carrillo, B., & Mendes, W. B. (2021). Is vicarious stress functionally adaptive? Perspective-taking modulates the effects of vicarious stress on future firsthand stress. *Emotion*. [https://doi.org/10.1037/emo0000963](https://psycnet.apa.org/doi/10.1037/emo0000963)

Stern, C., & West, T. V. (2014). Circumventing anxiety during interpersonal encounters to promote interest in contact: An implementation intention approach. *Journal of Experimental Social Psychology*, *50*, 82-93. <https://doi.org/10.1016/j.jesp.2013.09.008>

Turner, M. J., Jones, M. V., Sheffield, D., Barker, J. B., & Coffee, P. (2014). Manipulating cardiovascular indices of challenge and threat using resource appraisals. *International Journal of Psychophysiology*, *94*(1), 9-18. <https://doi.org/10.1016/j.ijpsycho.2014.07.004>

Turner, M. J., Jones, M. V., Sheffield, D., & Cross, S. L. (2012). Cardiovascular indices of challenge and threat states predict competitive performance. *International Journal of Psychophysiology*, *86*(1), 48-57. <https://doi.org/10.1016/j.ijpsycho.2012.08.004>

Table S1.

*Inter-correlations among key study variables*


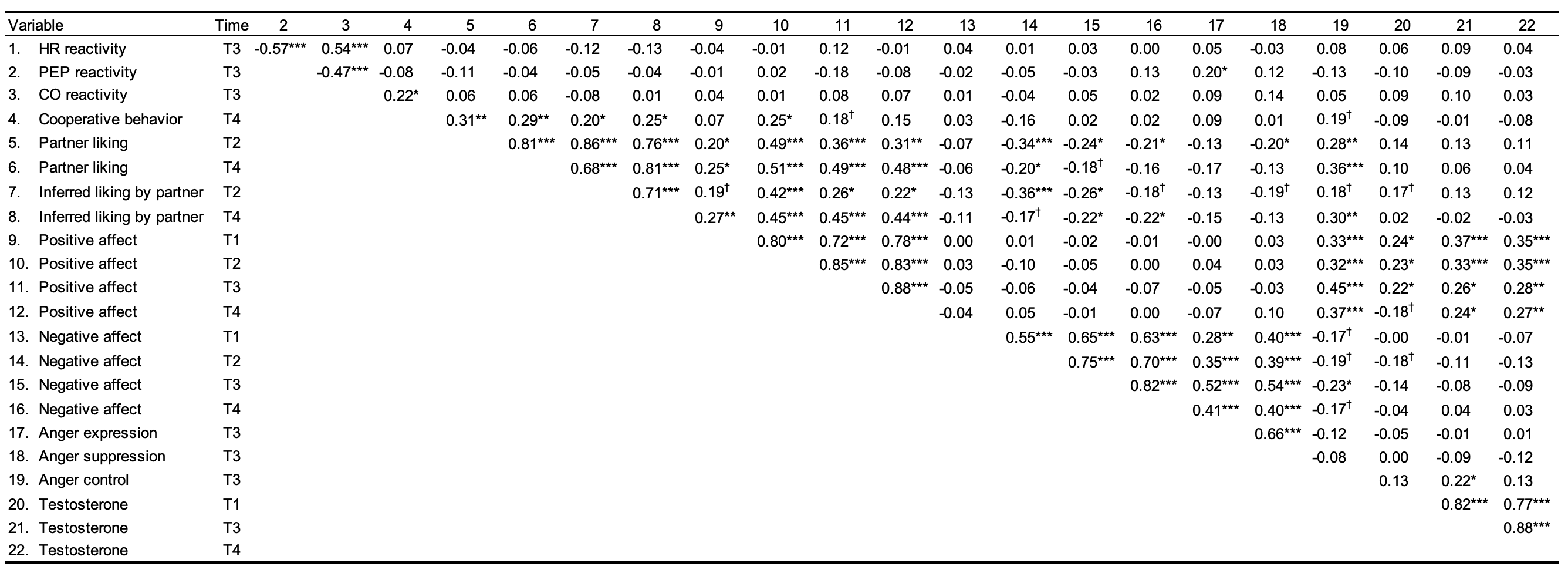


*Note*. Time column indicates assessment time of each variable; T1 (baseline), T2 (before the in-person interaction), T3 (during the in-person interaction), and T4 (after the in-person interaction). Cooperative behavior and testosterone responses are the raw data before transformation. The second and third testosterone samples were obtained at 18 and 33 minutes following the beginning of the in-person interaction, respectively. ^†^*p* < .10, **p* < .05, ***p* < .01, ****p* < .001.
